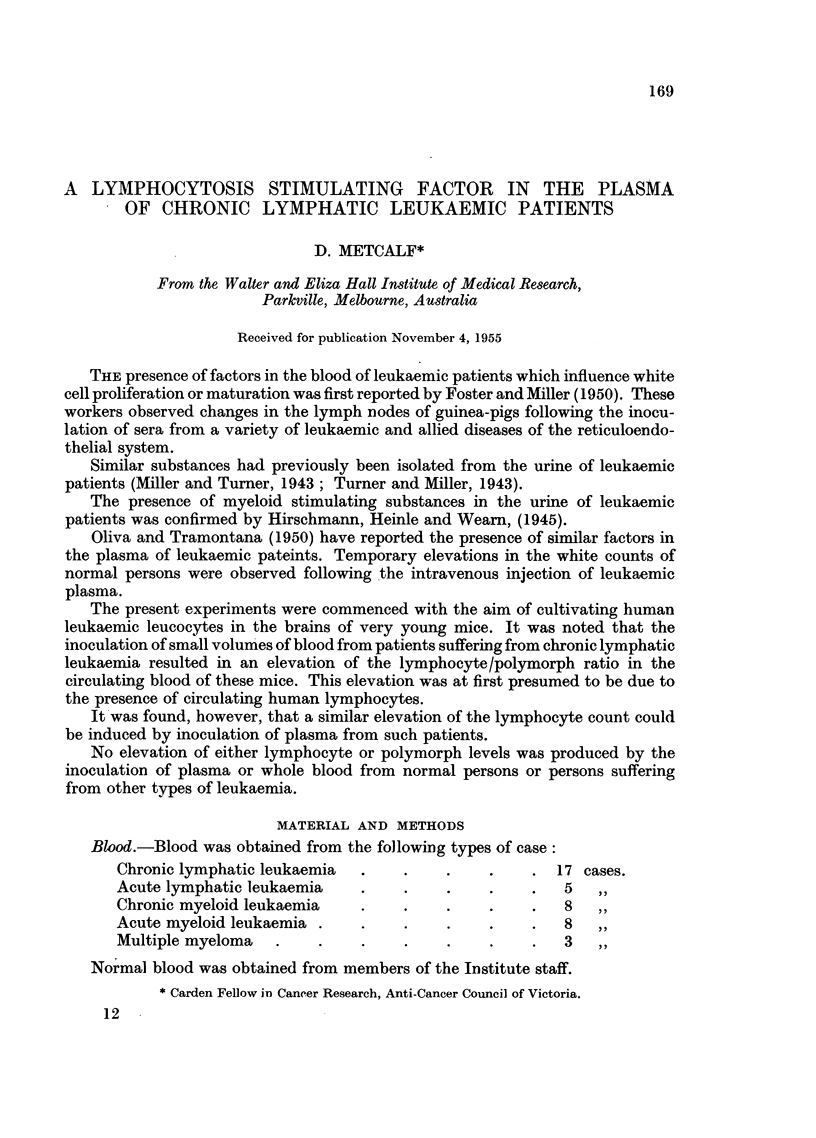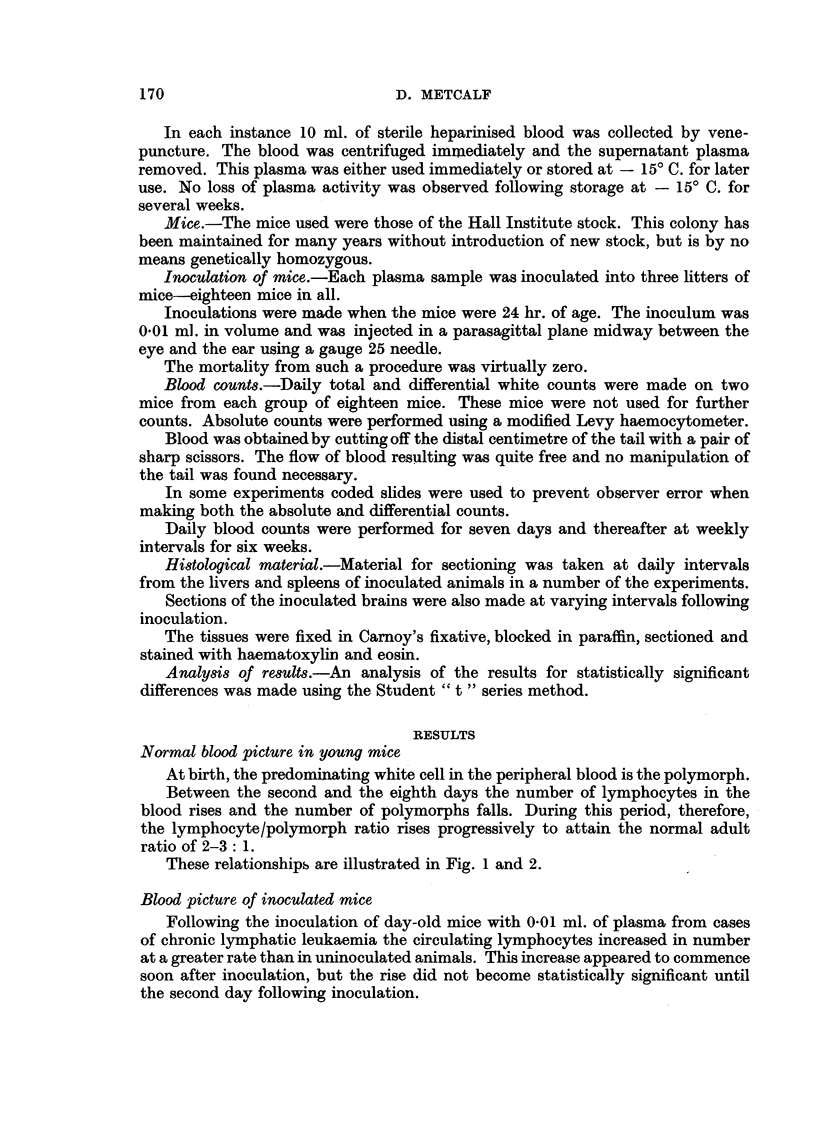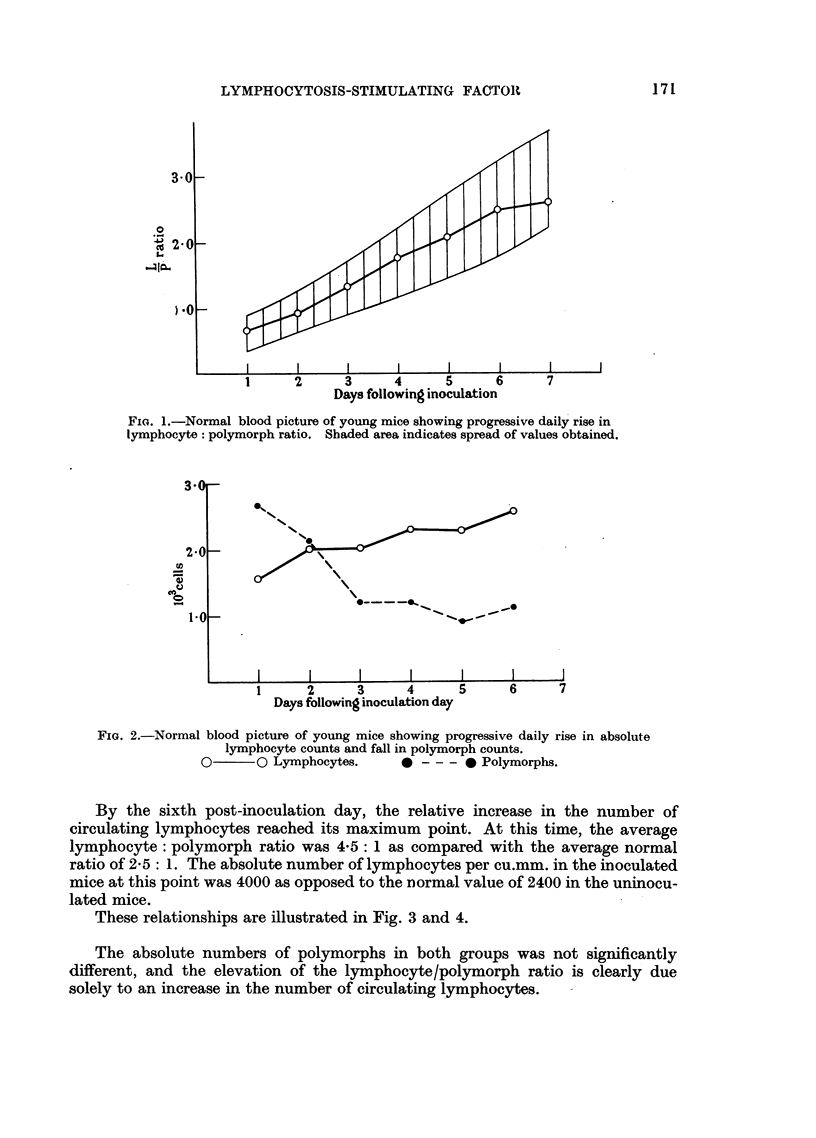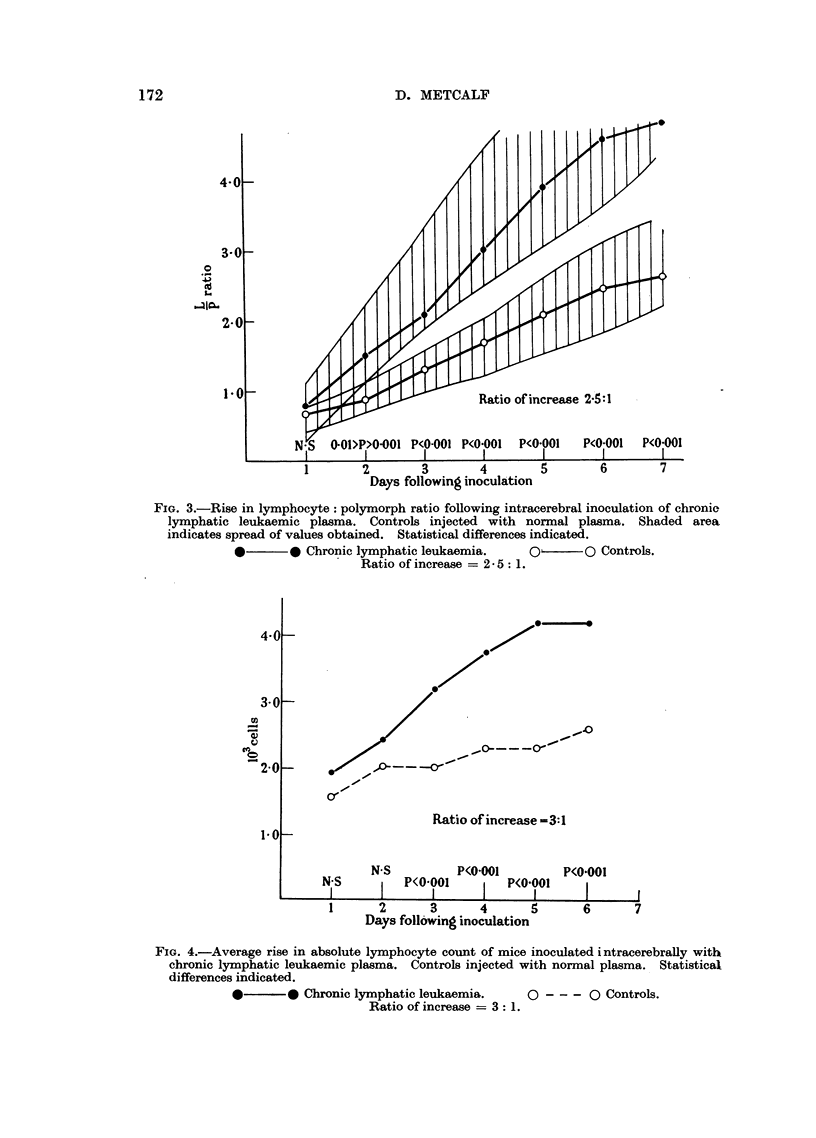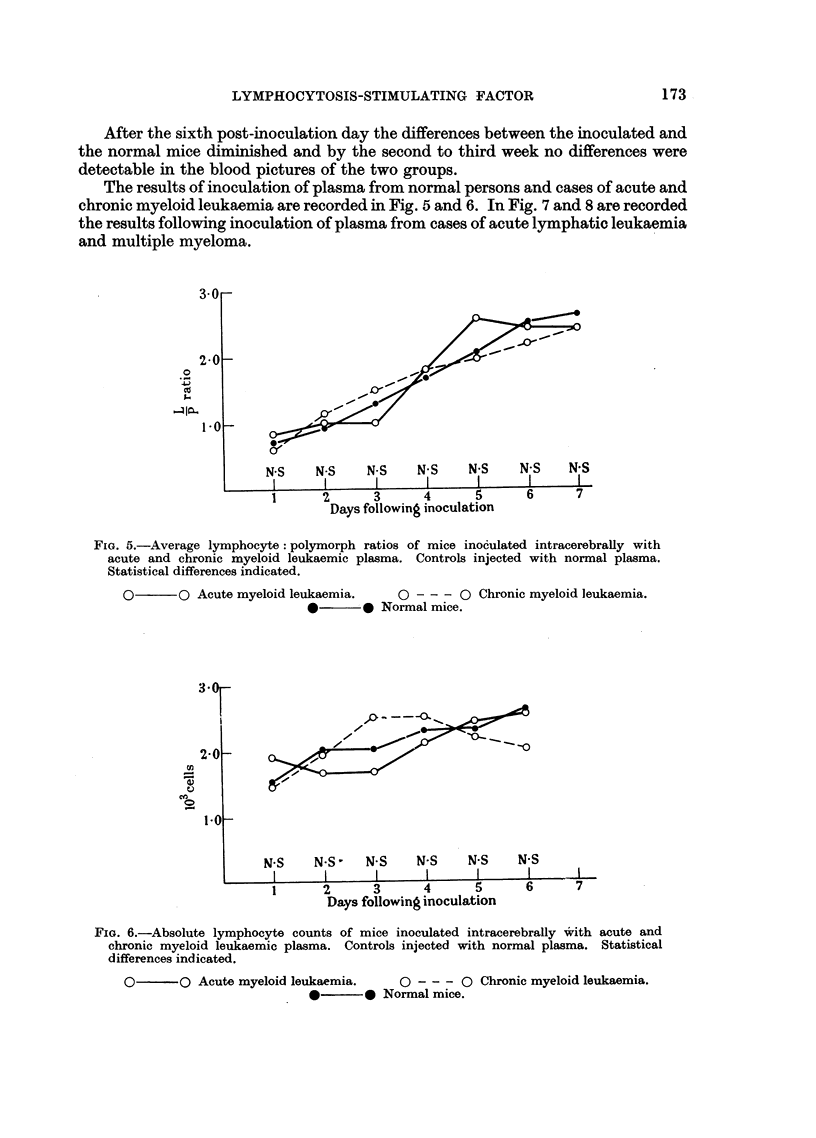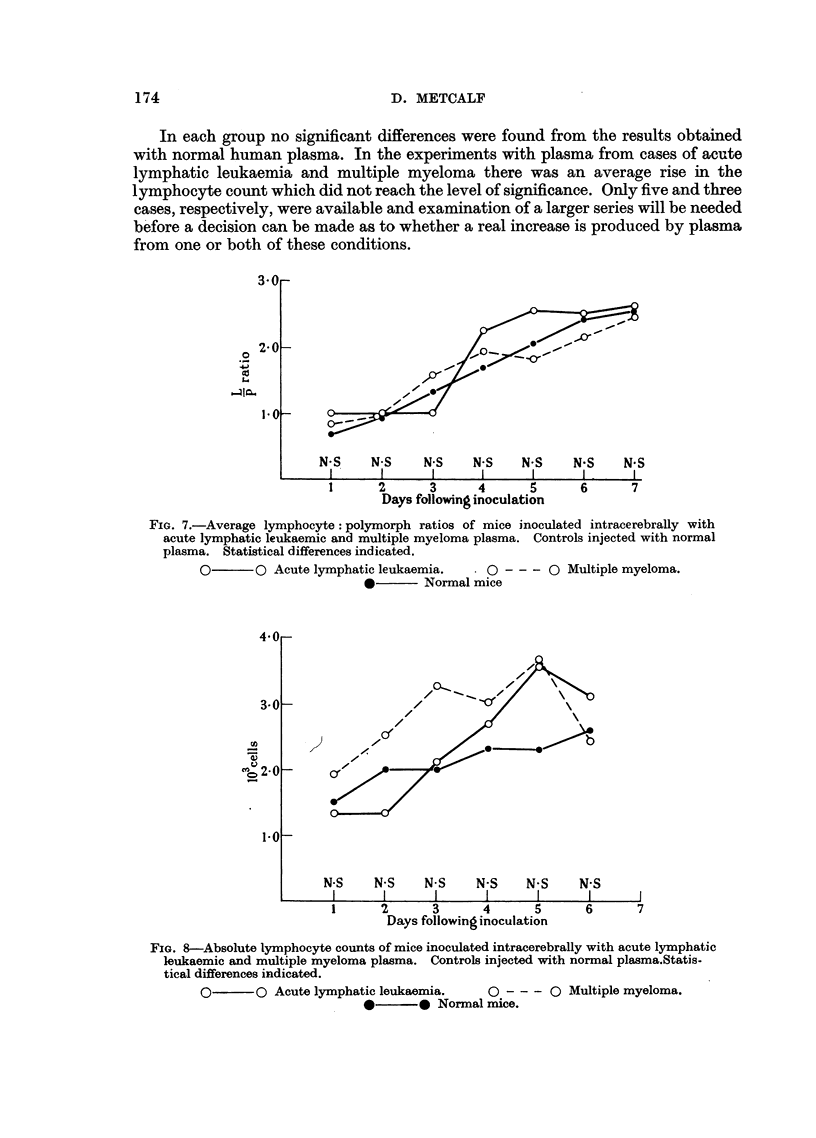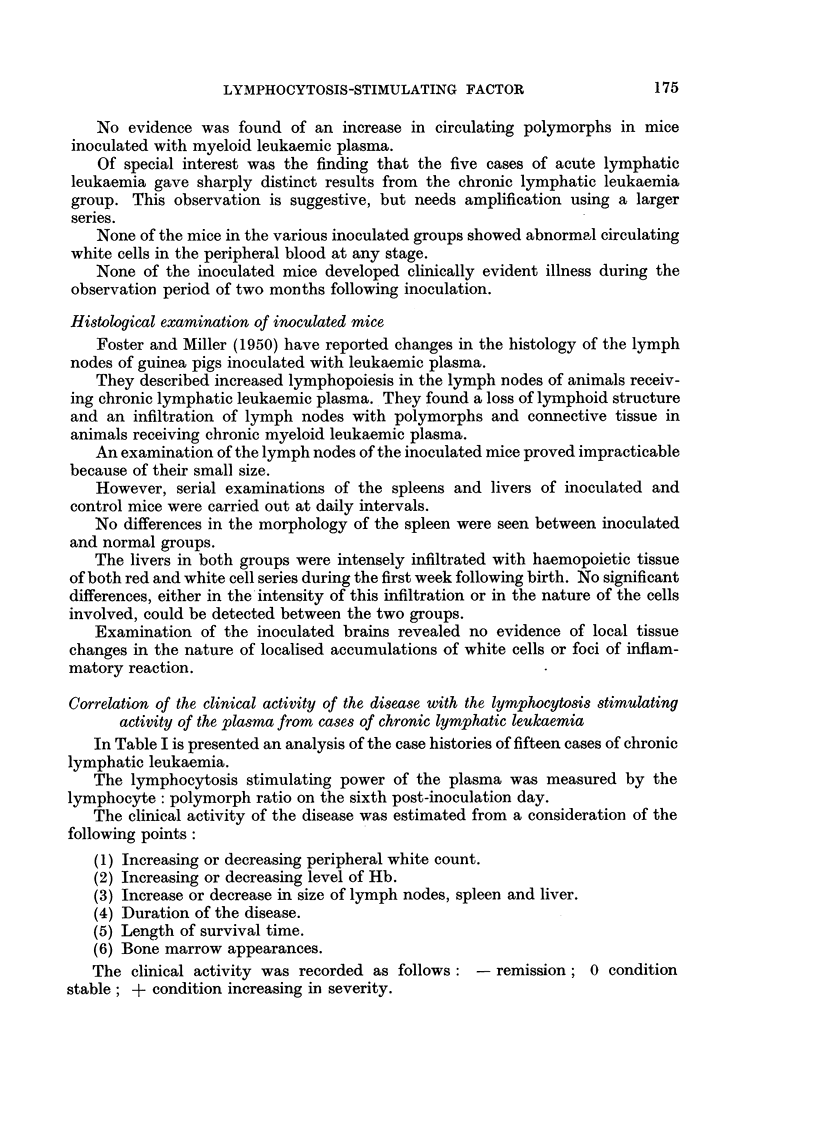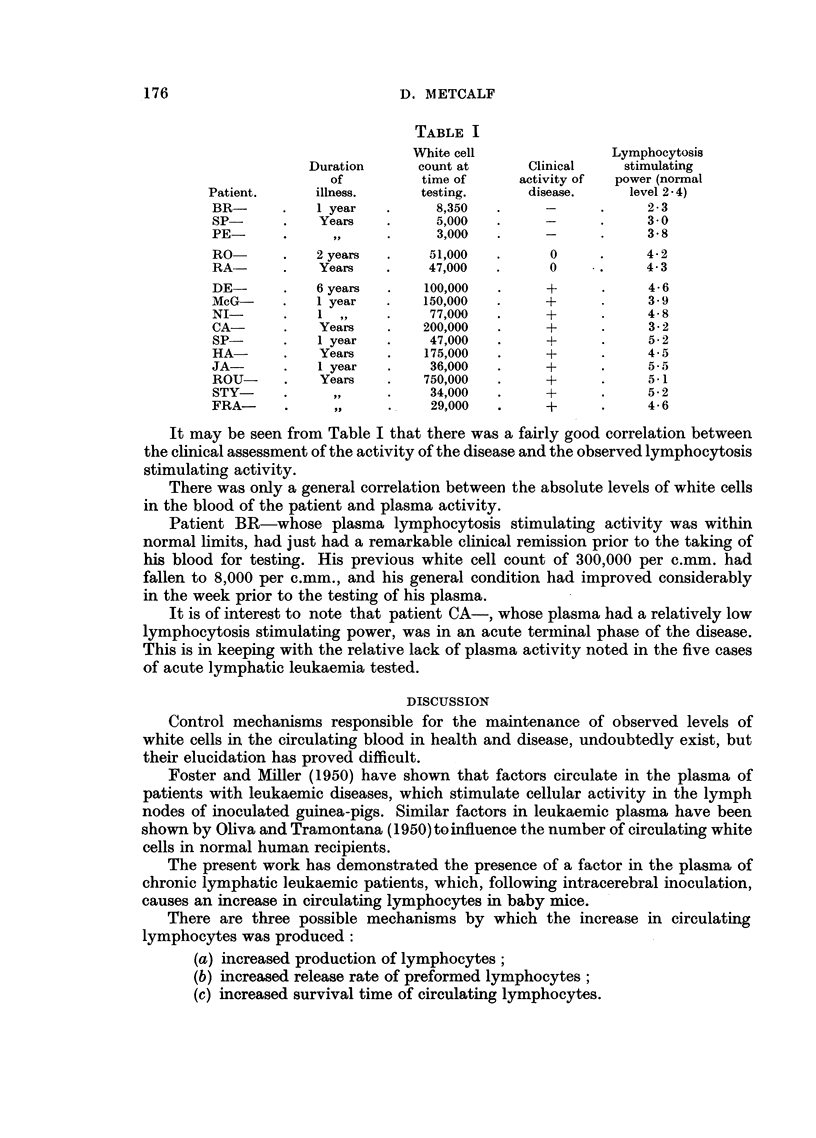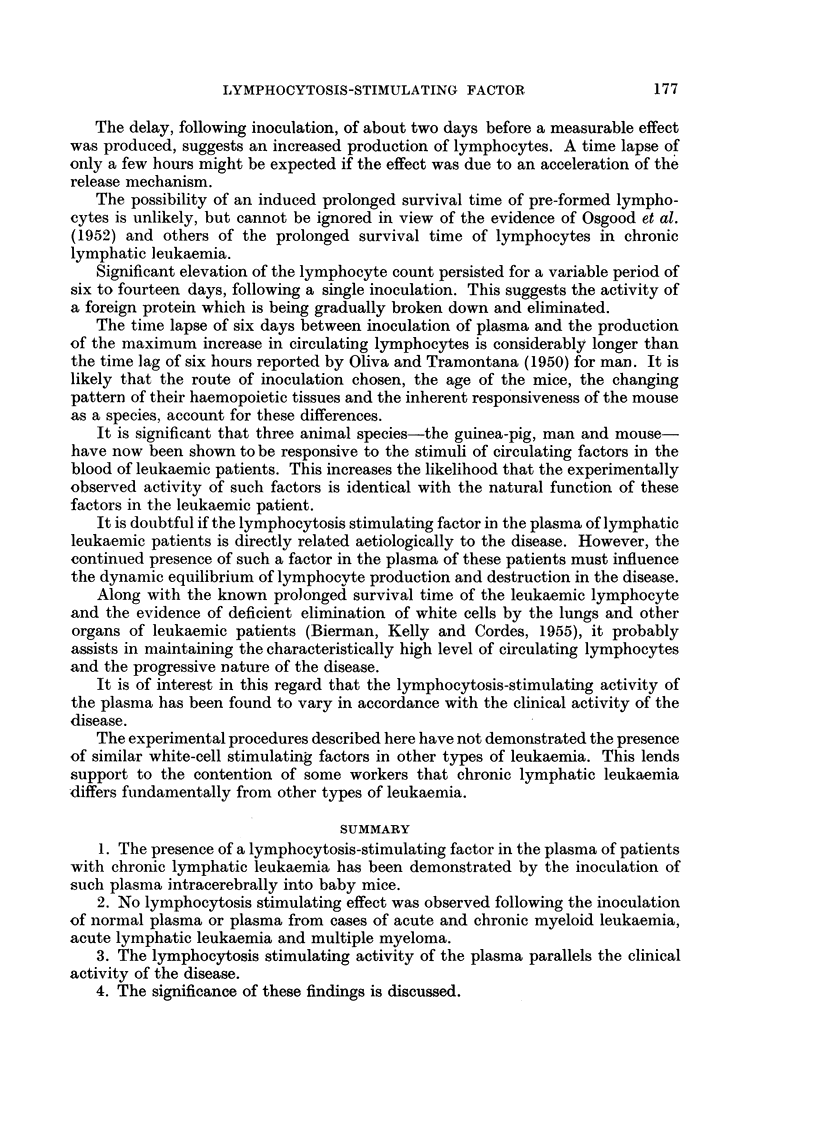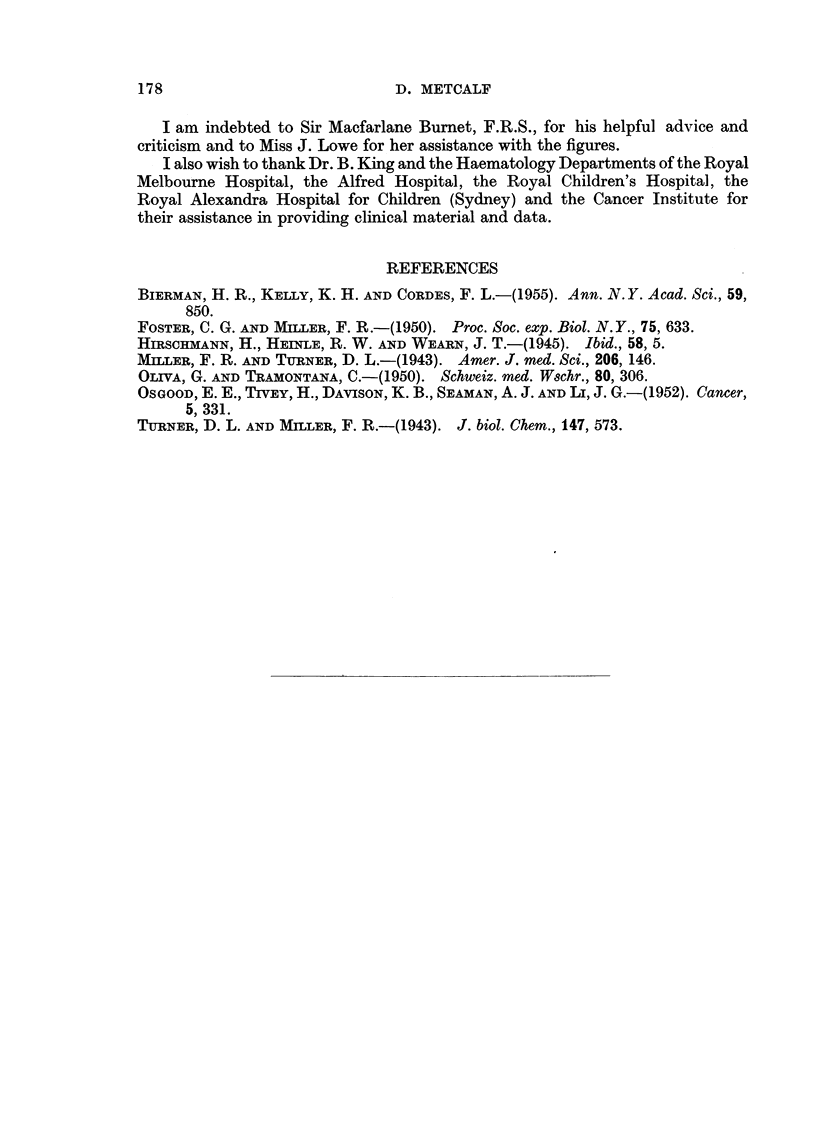# A Lymphocytosis Stimulating Factor in the Plasma of Chronic Lymphatic Leukaemic Patients

**DOI:** 10.1038/bjc.1956.20

**Published:** 1956-03

**Authors:** D. Metcalf


### A LYMPHOCYTOSIS STIMULATING FACTOR IN THE PLASMA OF CHRONIC LYMPHATIC LEUKAEMIC PATIENTS

D. METCALF*

*From the Walter and Eliza Hall Institute of Medical Research, Parkville, Melbourne, Australia*

Received for publication November 4, 1955

THE presence of factors in the blood of leukaemic patients which influence white cell proliferation or maturation was first reported by Foster and Miller (1950). These workers observed changes in the lymph nodes of guinea-pigs following the inoculation of sera from a variety of leukaemic and allied diseases of the reticuloendothelial system.

Similar substances had previously been isolated from the urine of leukaemic patients (Miller and Turner, 1943 ; Turner and Miller, 1943).

The presence of myeloid stimulating substances in the urine of leukaemic patients was confirmed by Hirschmann, Heinle and Wearn, (1945).

Oliva and Tramontana (1950) have reported the presence of similar factors in the plasma of leukaemic pateints. Temporary elevations in the white counts of normal persons were observed following the intravenous injection of leukaemic plasma.

The present experiments were commenced with the aim of cultivating human leukaemic leucocytes in the brains of very young mice. It was noted that the inoculation of small volumes of blood from patients suffering from chronic lymphatic leukaemia resulted in an elevation of the lymphocyte/polymorph ratio in the circulating blood of these mice. This elevation was at first presumed to be due to the presence of circulating human lymphocytes.

It was found, however, that a similar elevation of the lymphocyte count could be induced by inoculation of plasma from such patients.

No elevation of either lymphocyte or polymorph levels was produced by the inoculation of plasma or whole blood from normal persons or persons suffering from other types of leukaemia.

### MATERIAL AND METHODS

*Blood.*—Blood was obtained from the following types of case :

| | | |
|---|---|---|
| Chronic lymphatic leukaemia . . . . . | 17 | cases. |
| Acute lymphatic leukaemia . . . . . | 5 | ,, |
| Chronic myeloid leukaemia . . . . . | 8 | ,, |
| Acute myeloid leukaemia . . . . . . | 8 | ,, |
| Multiple myeloma . . . . . . . . | 3 | ,, |

Normal blood was obtained from members of the Institute staff.

* Carden Fellow in Cancer Research, Anti-Cancer Council of Victoria.

In each instance 10 ml. of sterile heparinised blood was collected by venepuncture. The blood was centrifuged immediately and the supernatant plasma removed. This plasma was either used immediately or stored at — 15° C. for later use. No loss of plasma activity was observed following storage at — 15° C. for several weeks.

*Mice.*—The mice used were those of the Hall Institute stock. This colony has been maintained for many years without introduction of new stock, but is by no means genetically homozygous.

*Inoculation of mice.*—Each plasma sample was inoculated into three litters of mice—eighteen mice in all.

Inoculations were made when the mice were 24 hr. of age. The inoculum was 0·01 ml. in volume and was injected in a parasagittal plane midway between the eye and the ear using a gauge 25 needle.

The mortality from such a procedure was virtually zero.

*Blood counts.*—Daily total and differential white counts were made on two mice from each group of eighteen mice. These mice were not used for further counts. Absolute counts were performed using a modified Levy haemocytometer.

Blood was obtained by cutting off the distal centimetre of the tail with a pair of sharp scissors. The flow of blood resulting was quite free and no manipulation of the tail was found necessary.

In some experiments coded slides were used to prevent observer error when making both the absolute and differential counts.

Daily blood counts were performed for seven days and thereafter at weekly intervals for six weeks.

*Histological material.*—Material for sectioning was taken at daily intervals from the livers and spleens of inoculated animals in a number of the experiments.

Sections of the inoculated brains were also made at varying intervals following inoculation.

The tissues were fixed in Carnoy's fixative, blocked in paraffin, sectioned and stained with haematoxylin and eosin.

*Analysis of results.*—An analysis of the results for statistically significant differences was made using the Student " t " series method.

### RESULTS

### *Normal blood picture in young mice*

At birth, the predominating white cell in the peripheral blood is the polymorph.

Between the second and the eighth days the number of lymphocytes in the blood rises and the number of polymorphs falls. During this period, therefore, the lymphocyte/polymorph ratio rises progressively to attain the normal adult ratio of 2–3 : 1.

These relationships are illustrated in Fig. 1 and 2.

### *Blood picture of inoculated mice*

Following the inoculation of day-old mice with 0·01 ml. of plasma from cases of chronic lymphatic leukaemia the circulating lymphocytes increased in number at a greater rate than in uninoculated animals. This increase appeared to commence soon after inoculation, but the rise did not become statistically significant until the second day following inoculation.

FIG. 1.—Normal blood picture of young mice showing progressive daily rise in lymphocyte : polymorph ratio. Shaded area indicates spread of values obtained.

FIG. 2.—Normal blood picture of young mice showing progressive daily rise in absolute lymphocyte counts and fall in polymorph counts.
○———○ Lymphocytes. ● – – – ● Polymorphs.

By the sixth post-inoculation day, the relative increase in the number of circulating lymphocytes reached its maximum point. At this time, the average lymphocyte : polymorph ratio was 4·5 : 1 as compared with the average normal ratio of 2·5 : 1. The absolute number of lymphocytes per cu.mm. in the inoculated mice at this point was 4000 as opposed to the normal value of 2400 in the uninoculated mice.

These relationships are illustrated in Fig. 3 and 4.

The absolute numbers of polymorphs in both groups was not significantly different, and the elevation of the lymphocyte/polymorph ratio is clearly due solely to an increase in the number of circulating lymphocytes.

FIG. 3.—Rise in lymphocyte : polymorph ratio following intracerebral inoculation of chronic lymphatic leukaemic plasma. Controls injected with normal plasma. Shaded area indicates spread of values obtained. Statistical differences indicated.

●———● Chronic lymphatic leukaemia. ○———○ Controls.
Ratio of increase = 2·5 : 1.

FIG. 4.—Average rise in absolute lymphocyte count of mice inoculated intracerebrally with chronic lymphatic leukaemic plasma. Controls injected with normal plasma. Statistical differences indicated.

●———● Chronic lymphatic leukaemia. ○ – – – ○ Controls.
Ratio of increase = 3 : 1.

After the sixth post-inoculation day the differences between the inoculated and the normal mice diminished and by the second to third week no differences were detectable in the blood pictures of the two groups.

The results of inoculation of plasma from normal persons and cases of acute and chronic myeloid leukaemia are recorded in Fig. 5 and 6. In Fig. 7 and 8 are recorded the results following inoculation of plasma from cases of acute lymphatic leukaemia and multiple myeloma.

Fig. 5.—Average lymphocyte : polymorph ratios of mice inoculated intracerebrally with acute and chronic myeloid leukaemic plasma. Controls injected with normal plasma. Statistical differences indicated.

○——○ Acute myeloid leukaemia. ○ - - - ○ Chronic myeloid leukaemia.

●——● Normal mice.

Fig. 6.—Absolute lymphocyte counts of mice inoculated intracerebrally with acute and chronic myeloid leukaemic plasma. Controls injected with normal plasma. Statistical differences indicated.

○——○ Acute myeloid leukaemia. ○ - - - ○ Chronic myeloid leukaemia.

●——● Normal mice.

In each group no significant differences were found from the results obtained with normal human plasma. In the experiments with plasma from cases of acute lymphatic leukaemia and multiple myeloma there was an average rise in the lymphocyte count which did not reach the level of significance. Only five and three cases, respectively, were available and examination of a larger series will be needed before a decision can be made as to whether a real increase is produced by plasma from one or both of these conditions.

Fig. 7.—Average lymphocyte : polymorph ratios of mice inoculated intracerebrally with acute lymphatic leukaemic and multiple myeloma plasma. Controls injected with normal plasma. Statistical differences indicated.

○——○ Acute lymphatic leukaemia. ○ – – – ○ Multiple myeloma.
●—— Normal mice

Fig. 8—Absolute lymphocyte counts of mice inoculated intracerebrally with acute lymphatic leukaemic and multiple myeloma plasma. Controls injected with normal plasma.Statistical differences indicated.

○——○ Acute lymphatic leukaemia. ○ – – – ○ Multiple myeloma.
●——● Normal mice.

No evidence was found of an increase in circulating polymorphs in mice inoculated with myeloid leukaemic plasma.

Of special interest was the finding that the five cases of acute lymphatic leukaemia gave sharply distinct results from the chronic lymphatic leukaemia group. This observation is suggestive, but needs amplification using a larger series.

None of the mice in the various inoculated groups showed abnormal circulating white cells in the peripheral blood at any stage.

None of the inoculated mice developed clinically evident illness during the observation period of two months following inoculation.

*Histological examination of inoculated mice*

Foster and Miller (1950) have reported changes in the histology of the lymph nodes of guinea pigs inoculated with leukaemic plasma.

They described increased lymphopoiesis in the lymph nodes of animals receiving chronic lymphatic leukaemic plasma. They found a loss of lymphoid structure and an infiltration of lymph nodes with polymorphs and connective tissue in animals receiving chronic myeloid leukaemic plasma.

An examination of the lymph nodes of the inoculated mice proved impracticable because of their small size.

However, serial examinations of the spleens and livers of inoculated and control mice were carried out at daily intervals.

No differences in the morphology of the spleen were seen between inoculated and normal groups.

The livers in both groups were intensely infiltrated with haemopoietic tissue of both red and white cell series during the first week following birth. No significant differences, either in the intensity of this infiltration or in the nature of the cells involved, could be detected between the two groups.

Examination of the inoculated brains revealed no evidence of local tissue changes in the nature of localised accumulations of white cells or foci of inflammatory reaction.

*Correlation of the clinical activity of the disease with the lymphocytosis stimulating activity of the plasma from cases of chronic lymphatic leukaemia*

In Table I is presented an analysis of the case histories of fifteen cases of chronic lymphatic leukaemia.

The lymphocytosis stimulating power of the plasma was measured by the lymphocyte : polymorph ratio on the sixth post-inoculation day.

The clinical activity of the disease was estimated from a consideration of the following points :

(1) Increasing or decreasing peripheral white count.
(2) Increasing or decreasing level of Hb.
(3) Increase or decrease in size of lymph nodes, spleen and liver.
(4) Duration of the disease.
(5) Length of survival time.
(6) Bone marrow appearances.

The clinical activity was recorded as follows : — remission ; 0 condition stable ; + condition increasing in severity.

TABLE I

| Patient. | Duration of illness. | White cell count at time of testing. | Clinical activity of disease. | Lymphocytosis stimulating power (normal level 2·4) |
|---|---|---|---|---|
| BR— | 1 year | 8,350 | — | 2·3 |
| SP— | Years | 5,000 | — | 3·0 |
| PE— | ,, | 3,000 | — | 3·8 |
| RO— | 2 years | 51,000 | 0 | 4·2 |
| RA— | Years | 47,000 | 0 | 4·3 |
| DE— | 6 years | 100,000 | + | 4·6 |
| McG— | 1 year | 150,000 | + | 3·9 |
| NI— | 1 ,, | 77,000 | + | 4·8 |
| CA— | Years | 200,000 | + | 3·2 |
| SP— | 1 year | 47,000 | + | 5·2 |
| HA— | Years | 175,000 | + | 4·5 |
| JA— | 1 year | 36,000 | + | 5·5 |
| ROU— | Years | 750,000 | + | 5·1 |
| STY— | ,, | 34,000 | + | 5·2 |
| FRA— | ,, | 29,000 | + | 4·6 |

It may be seen from Table I that there was a fairly good correlation between the clinical assessment of the activity of the disease and the observed lymphocytosis stimulating activity.

There was only a general correlation between the absolute levels of white cells in the blood of the patient and plasma activity.

Patient BR—whose plasma lymphocytosis stimulating activity was within normal limits, had just had a remarkable clinical remission prior to the taking of his blood for testing. His previous white cell count of 300,000 per c.mm. had fallen to 8,000 per c.mm., and his general condition had improved considerably in the week prior to the testing of his plasma.

It is of interest to note that patient CA—, whose plasma had a relatively low lymphocytosis stimulating power, was in an acute terminal phase of the disease. This is in keeping with the relative lack of plasma activity noted in the five cases of acute lymphatic leukaemia tested.

### DISCUSSION

Control mechanisms responsible for the maintenance of observed levels of white cells in the circulating blood in health and disease, undoubtedly exist, but their elucidation has proved difficult.

Foster and Miller (1950) have shown that factors circulate in the plasma of patients with leukaemic diseases, which stimulate cellular activity in the lymph nodes of inoculated guinea-pigs. Similar factors in leukaemic plasma have been shown by Oliva and Tramontana (1950) to influence the number of circulating white cells in normal human recipients.

The present work has demonstrated the presence of a factor in the plasma of chronic lymphatic leukaemic patients, which, following intracerebral inoculation, causes an increase in circulating lymphocytes in baby mice.

There are three possible mechanisms by which the increase in circulating lymphocytes was produced :

(*a*) increased production of lymphocytes ;
(*b*) increased release rate of preformed lymphocytes ;
(*c*) increased survival time of circulating lymphocytes.

The delay, following inoculation, of about two days before a measurable effect was produced, suggests an increased production of lymphocytes. A time lapse of only a few hours might be expected if the effect was due to an acceleration of the release mechanism.

The possibility of an induced prolonged survival time of pre-formed lymphocytes is unlikely, but cannot be ignored in view of the evidence of Osgood *et al.* (1952) and others of the prolonged survival time of lymphocytes in chronic lymphatic leukaemia.

Significant elevation of the lymphocyte count persisted for a variable period of six to fourteen days, following a single inoculation. This suggests the activity of a foreign protein which is being gradually broken down and eliminated.

The time lapse of six days between inoculation of plasma and the production of the maximum increase in circulating lymphocytes is considerably longer than the time lag of six hours reported by Oliva and Tramontana (1950) for man. It is likely that the route of inoculation chosen, the age of the mice, the changing pattern of their haemopoietic tissues and the inherent responsiveness of the mouse as a species, account for these differences.

It is significant that three animal species—the guinea-pig, man and mouse—have now been shown to be responsive to the stimuli of circulating factors in the blood of leukaemic patients. This increases the likelihood that the experimentally observed activity of such factors is identical with the natural function of these factors in the leukaemic patient.

It is doubtful if the lymphocytosis stimulating factor in the plasma of lymphatic leukaemic patients is directly related aetiologically to the disease. However, the continued presence of such a factor in the plasma of these patients must influence the dynamic equilibrium of lymphocyte production and destruction in the disease.

Along with the known prolonged survival time of the leukaemic lymphocyte and the evidence of deficient elimination of white cells by the lungs and other organs of leukaemic patients (Bierman, Kelly and Cordes, 1955), it probably assists in maintaining the characteristically high level of circulating lymphocytes and the progressive nature of the disease.

It is of interest in this regard that the lymphocytosis-stimulating activity of the plasma has been found to vary in accordance with the clinical activity of the disease.

The experimental procedures described here have not demonstrated the presence of similar white-cell stimulating factors in other types of leukaemia. This lends support to the contention of some workers that chronic lymphatic leukaemia differs fundamentally from other types of leukaemia.

### SUMMARY

1. The presence of a lymphocytosis-stimulating factor in the plasma of patients with chronic lymphatic leukaemia has been demonstrated by the inoculation of such plasma intracerebrally into baby mice.

2. No lymphocytosis stimulating effect was observed following the inoculation of normal plasma or plasma from cases of acute and chronic myeloid leukaemia, acute lymphatic leukaemia and multiple myeloma.

3. The lymphocytosis stimulating activity of the plasma parallels the clinical activity of the disease.

4. The significance of these findings is discussed.

I am indebted to Sir Macfarlane Burnet, F.R.S., for his helpful advice and criticism and to Miss J. Lowe for her assistance with the figures.

I also wish to thank Dr. B. King and the Haematology Departments of the Royal Melbourne Hospital, the Alfred Hospital, the Royal Children's Hospital, the Royal Alexandra Hospital for Children (Sydney) and the Cancer Institute for their assistance in providing clinical material and data.

### REFERENCES

BIERMAN, H. R., KELLY, K. H. AND CORDES, F. L.—(1955). *Ann. N.Y. Acad. Sci.*, **59**, 850.

FOSTER, C. G. AND MILLER, F. R.—(1950). *Proc. Soc. exp. Biol. N.Y.*, **75**, 633.

HIRSCHMANN, H., HEINLE, R. W. AND WEARN, J. T.—(1945). *Ibid.*, **58**, 5.

MILLER, F. R. AND TURNER, D. L.—(1943). *Amer. J. med. Sci.*, **206**, 146.

OLIVA, G. AND TRAMONTANA, C.—(1950). *Schweiz. med. Wschr.*, **80**, 306.

OSGOOD, E. E., TIVEY, H., DAVISON, K. B., SEAMAN, A. J. AND LI, J. G.—(1952). *Cancer*, **5**, 331.

TURNER, D. L. AND MILLER, F. R.—(1943). *J. biol. Chem.*, **147**, 573.